# The dosage of thiopental as pharmacological cerebral protection during non-shunt carotid endarterectomy: A retrospective study

**DOI:** 10.12688/f1000research.131838.3

**Published:** 2023-12-11

**Authors:** Pimwan Sookplung, Pathomporn Suchartwatnachai, Phuping Akavipat

**Affiliations:** 1 Department of Anesthesiology, Neurological Institute of Thailand, Bangkok, 10400, Thailand

**Keywords:** Carotid endarterectomy, barbiturate, thiopental, burst suppression, electroencephalogram

## Abstract

**Background:**

Thiopental has been used as a pharmacological cerebral protection strategy during carotid endarterectomy surgeries. However, the optimal dosage required to induce burst suppression on the electroencephalogram (EEG) remains unknown. This retrospective study aimed to determine the optimal dosage of thiopental required to induce burst suppression during non-shunt carotid endarterectomy.

**Methods:**

The Neurological Institute of Thailand Review Board approved the study. Data were collected from 2009 to 2019 for all non-shunt carotid endarterectomy patients who received thiopental for pharmacological cerebral protection and had intraoperative EEG monitoring. Demographic information, carotid stenosis severity, intraoperative EEG parameters, thiopental dosage, carotid clamp time, intraoperative events, and patient outcomes were abstracted.

**Results:**

The study included 57 patients. Among them, 24 patients (42%) achieved EEG burst suppression pattern with a thiopental dosage of 26.3±10.1 mg/kg/hr. There were no significant differences in perioperative events between patients who achieved burst suppression and those who did not. After surgery, 33.3% of patients who achieved burst suppression were extubated and awakened. One patient in the non-burst suppression group experienced mild neurological deficits. No deaths occurred within one month postoperative.

**Conclusions:**

The optimal dosage of thiopental required to achieve burst suppression on intraoperative EEG during non-shunt carotid endarterectomy was 26.3±10.1 mg/kg/hr.

## Introduction

Stroke is one of the leading causes of death and disability in Thailand, ^1^ and carotid stenosis is one of the leading causes of stroke. ^2^ The surgical treatment to prevent stroke is carotid endarterectomy (CEA). It is associated with periprocedural risks, including stroke (embolic or hemodynamic), myocardial infarction, and death. Therefore, strict selection criteria are applied for patients undergoing CEA. Current selection criteria support CEA for symptomatic low-risk surgical patients with 50% to 99% stenosis and asymptomatic patients with stenosis of 70% to 99%. ^3^ However, the ability of the patient to tolerate the cross-clamp depends on the sufficiency of collateral flow through the circle of Willis. Inadequate collateral cerebral perfusion during the cross-clamp period increases the risk of perioperative stroke. ^4^ Despite routine intraluminal shunt during the temporary occlusion of the ipsilateral internal carotid artery being controversial, intraoperative electrophysiological monitoring, such as electroencephalogram (EEG), is a valuable tool to detect cerebral hypoperfusion and determine selective shunting. ^5^^,^^6^ When the neurosurgeon performed the non-shunt technique, adequate cerebral perfusion during carotid cross-clamping could be achieved using several methods to protect the brain. ^7^ Spetzler et al. ^8^ reported excellent non-shunt surgical outcomes using intra-operative barbiturate and microsurgical techniques. The clinical use of barbiturates is known for cerebral protection against the prevention of focal cerebral ischemia, ^9^ especially when barbiturate was administered before the ischemic insult with doses large enough to produce burst-suppression activity on the EEG. ^10^

Despite the lack of clarity, the rationale for inducing burst suppression is based on its theorized potential neuroprotective effects. Burst suppression reduces metabolic demand by decreasing intracellular adenosine triphosphate (ATP) concentration, leading to decreased neuronal activity and electrical signaling, ^11^ reduction in cerebral blood flow (CBF), ^12^ and preserving limited energy resources during critical situations. Although, experimental animal studies conducted by Warner DS ^13^ and Robert Schmid-Elsaesser, ^14^ indicate that EEG burst suppression may not be necessary for maximum cerebral protection. Additionally, Westermaier T ^15^ found no additional neuroprotection following mild hypothermic treatment of rats subjected to reversible focal ischemia by barbiturate-induced burst suppression. Despite this, animal studies may not be conclusive in humans due to inter-species differences in burst suppression effects ^16^ and differences in physiology and the human clinical context.

Human research is crucial for understanding burst suppression benefits in clinical settings. Doyle PW ^17^ suggests that if the flow-metabolism coupling is intact, complete EEG burst suppression (100% burst suppression) may provide more cerebral protection than 50% burst suppression. However, the study did not evaluate the cerebral protection effects. Thus, further human studies are still needed to fully understand the relationship between burst suppression and cerebral protection, also the definite predetermined amount of barbiturate-induced burst-suppression activity on EEG including the optimal dosage, timing, and administration mode varies among studies to reach the burst-suppression pattern.

The Neurological Institute of Thailand is one of the few medical centers with EEG for intraoperative surveillance. Thus, to fill the knowledge gap, the authors aimed to study the optimal dose of barbiturates as thiopental for inducing EEG burst-suppression patterns in anesthetized patients undergoing carotid endarterectomy with a non-shunt technique.

## Methods

### Study design and participants

The study was approved by The Research Ethics Committee of the Neurological Institute of Thailand (approval number IRB53068). Written informed consent was waived, as this study was a retrospective observational without patient interventions. Data were collected from all consecutive patients with carotid artery stenosis who underwent CEA at the Neurological Institute of Thailand, Bangkok, from January 2009 to December 2019. Patients scheduled for CEA with intraoperative EEG were included, while Patients undergoing CEA without thiopental as pharmacological cerebral protection were excluded.

### Pre-operative investigation

Patients with prior minor stroke, reversible ischemic neurological deficit (RIND), or transient ischemic attack (TIA) underwent Computed tomography (CT) brain scans. Carotid duplex ultrasound and magnetic resonance angiography (MRA) assessed carotid disease, degree of stenosis, and collateral circulation. Some cases had additional angiography to evaluate the carotid disease and the collateral circulation status. Carotid endarterectomy was based on duplex and MRA results. Patients with poor collateral flow had specific intraoperative assessment using the "Backflow technique." Insufficient blood flow led to intraluminal shunt use during carotid clamping.

### Management of anesthesia and perioperative care

According to the institution, our neurosurgeons prefer non-shunt carotid endarterectomy under general anesthesia with pharmacological cerebral protection strategies and an increased blood pressure of 10% to promote collateral circulation and prevent ischemic complications during carotid clamping. ^18^

### Monitoring of burst-suppression patterns by EEG

In addition to standard anesthetic monitoring with an arterial line, all patients were monitored with the two-channel cortical EEG using the EEG pod of Infinity Delta Series (Drager Medical AG & Co. Lubeck, Germany). The EEG signal was obtained using silver-silver chloride electrodes located according to the international 10-20 systems. The differential montage was recorded: left and right frontal (FP1-C3, FP2-C4; channels 1 and 2), with a neutral electrode placed at the ear lobe. The impedance was recommended at < 5,000 ohms. Power Spectra analysis (Fast Fourier transform: FFT) was used to simplify the complex EEG to computer-processed EEG (CEEG) for an 8-second epoch. Trained anesthesiologists visually assessed the raw EEG and compressed spectral EEG parameters [Spectral Edge Frequency 95% (SEF95%), Median (MED), and Burst Suppression Ratio (BSR)]. Burst Suppression Ratio (BSR) was defined as the percentage of time the EEG waveform is flatlined over the last 60 seconds (when flatline EEG alternates with “bursts” of activity).

### Anesthetic procedures

Anesthesia was induced with thiopental (Pentothal Sodium ^®^) (3-5 mg/kg) or propofol (1-2 mg/kg), followed by fentanyl (1-2 mcg/kg), atracurium (0.5-0.6 mg/kg), or cis-atracurium (0.15 mg/kg) to facilitate tracheal intubation. The anesthesia was maintained with sevoflurane or desflurane (<1 MAC) and continuous infusion of a neuromuscular blocking agent (atracurium 0.3-0.5 mg/kg/hr or cis-atracurium 0.06-0.1 mg/kg/hr). An additional dose of fentanyl 25-50 mcg was titrated during the operation. Antihypertensive medications were administered for hypertension, and fluids or vasopressors were used to treat hypotension.

Before the temporary occlusion of the carotid artery, a single dose of heparin 5,000 units and Thiopental (5 mg/kg) was given intravenously (IV), followed by continuous infusion of 10 mg/kg/hr. An additional 50 mg was titrated intravenously by the anesthesiologist's decision, with the timing varying to achieve burst suppression on EEG throughout the ischemic period. There are no specific guidelines regarding how often the additional dose of thiopental should be administered. During the carotid clamp time, the blood pressure was raised 10% above the pre-operative level to induce collateral circulation. The inhalation agent was suspended during the thiopental infusion. At the end of the operation, the neuromuscular blockade was reversed with neostigmine 0.02 to 0.07 mg/kg combined with glycopyrrolate 0.2 mg or atropine 0.02 mg/kg. The patient was extubated if the patient had adequate ventilation, eye-opening, and purposeful responses. All patients were transferred to the neurosurgical intensive care unit for postoperative care.

### Data collection

Electronic database searches and manual data were abstracted, including demographic data, clinical courses, and outcomes. Patient characteristics were age, gender, American Society of Anesthesiologists Physical Status classification (ASA), Glasgow Coma Scale (GCS), and history of any comorbidities: cerebrovascular accident or transient ischemic attack (TIA), coronary artery disease (CAD), hypertension (HT), diabetes mellitus (DM), and dyslipidemia. Pre-operative investigation data such as the site and degree of stenosis measured by carotid duplex ultrasonography and magnetic resonance angiography (MRA) or conventional angiography were abstracted.

Intraoperative data including EEG parameters [Burst suppression ratio (BSR), Spectral edge frequency 95% (SEF95%), Median (MED)], thiopental dosage, carotid clamp time, intraoperative events (hypertension, hypotension, cardiac arrhythmias), duration of surgery, fluid administration, estimated blood loss, perioperative blood product transfusions (units), and successful extubation after surgery. Extubation time in the neurosurgical intensive care unit, Glasgow outcome scale (GOS) at discharge, and one-month postoperative were studied.

### Outcome measures

The study's primary outcome was the amount of thiopental required to achieve burst suppression on EEG during cerebral protection. In addition, the study investigated several secondary outcomes related to the patient's recovery, including the percentage of successful extubations after surgery, the time to extubation in the neurosurgical intensive care unit, and the Glasgow Outcome Scale (GOS) at discharge and one month postoperatively. These measures were analyzed to assess the patient's recovery following the procedure.

### Statistical analysis

Statistical analysis was performed using SPSS Statistical software, version 22 (IBM SPSS Inc., Chicago, IL). Descriptive statistics were presented as means±standard deviations, percentages, and numbers. The Chi-square test was used to compare categorical variables, while unpaired t-tests were employed for analyzing continuous variables. Paired t-tests were utilized to compare EEG data before and the average of EEG data during the carotid cross-clamp. A significance level of P-value≤0.05 was considered statistical significance.

## Results

**Figure 1.**
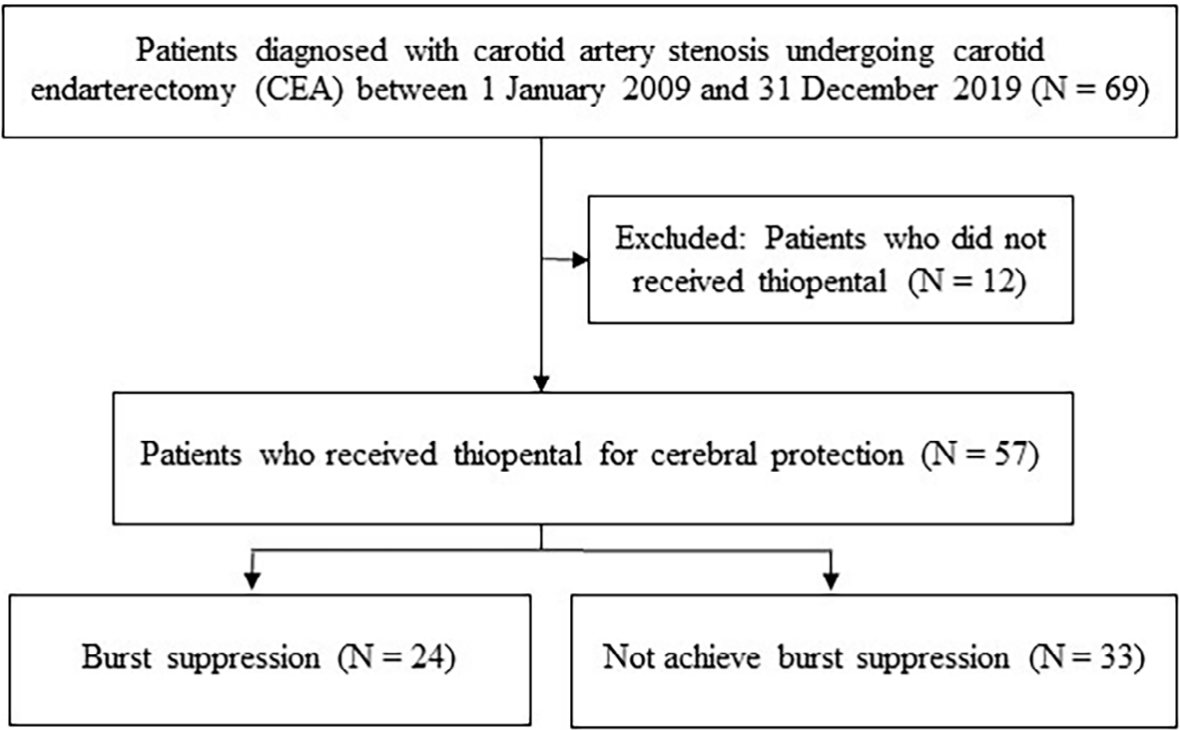
Flow diagram of the study.

There were 69 carotid endarterectomies performed during the study period, with 12 cases excluded. Of the remaining 57 patients analyzed ( Figure 1), only 24 achieved burst suppression on intraoperative EEG despite receiving continuous thiopental infusion with additional titration. These 24 patients were classified as the burst suppression group (BS) for the analysis. The demographic data and related details of both the BS and non-BS group are presented in Table 1. The group had a significantly higher average age of 72.8±9.1 years than the non-BS group, with an average age of 66.7±7.2 years (p-value=0.007). However, there were no significant differences in gender, body weight, ASA physical status, comorbidities, or pre-operative investigation data between the two groups. Hypertension was a common condition in both groups. The percentage of patients who received thiopental or propofol as induction agents and the dosages were not significantly different between the two groups ( Table 2). Perioperative doses of fentanyl and end-tidal concentrations of sevoflurane or desflurane also showed no significant differences. The amount of thiopental required to achieve burst suppression on intraoperative EEG was significantly higher in the BS group compared to the non-BS group (26.3±10.1 mg/kg/hr vs. 18.7±8.8, p-value=0.004). Although the carotid clamp time was slightly shorter in the BS group, it did not reach statistical significance (73.2±23.7 min vs. 83.3±34.8, p-value=0.225).

**Table 1.** Demographic data.

Variables	Group BS (n=24)	Group Non-BS (n=33)	p-value
Age (year)	72.8±9.1	66.7±7.2	0.007
Gender: Male	7 (29.2)	13 (39.4)	0.424
ASA II/III	21 (87.5)/3 (12.5)	22 (66.7)/11 (33.3)	0.071
Weight (kg)	64.6±10.4	62.5±7.4	0.397
Glasgow Coma Score=15	22 (91.7)	31 (93.9)	0.74
Comorbidities (n)			
Coronary Artery Disease	3 (12.5)	4 (12.1)	0.966
Hypertension	18 (75)	22 (66.7)	0.497
Diabetes Mellitus	12 (50)	16 (48.5)	0.910
Dyslipidemia	6 (25)	11 (33.3)	0.497
Pre-operative investigation data			
Bilateral stenosis	3 (12.5)	5 (15.2)	0.776
Site of operation Right/Left	15 (62.5)/9 (37.5)	21 (63.6)/12 (36.4)	0.930

The data are presented as mean±standard deviation or n (%), P value<0.05 indicates statistical significance.

**Table 2.** Perioperative data.

Variables	Group BS (n=24)	Group Non-BS (n=33)	p-value
Induction:			
Thiopental/Propofol	7 (29.2)/17 (70.8)	14 (42.4)/19 (57.6)	0.306
Thiopental (mg/kg)	3.9±0.3	4.7±0.7	0.087
Propofol (mg/kg)	1.5±0.4	1.3±1.2	0.547
During carotid clamp:			
Total thiopental (mg)	1,951.8±741.4	1,411.7±439.8	0.001
Total thiopental (mg/kg/hr)	26.3±10.1	18.7±8.8	0.004
Fentanyl (mcg/kg/hr)	0.9±0.3	1±0.3	0.838
Sevoflurane/Desflurane	15 (62.5)/9 (37.5)	24 (72.7)/9 (27.7)	0.296
Sevoflurane/Desflurane (% end tidal)	0.2±0.1/1.6±0.7	0.2±0.1/1.7±0.7	0.310/0.921
Carotid clamp time (min)	73.2±23.7	83.3±34.8	0.225
Operative time (min)	180.2±39.4	193.8±47.1	0.254
Anesthetic time (min)	243.8±43.6	251.1±51.1	0.576
Crystalloid (mL)	1,795.1±652.6	1,867.1±574.4	0.661
Estimated blood loss (mL)	141.9±175.1	172.6±187.7	0.533
Urine output (mL)	909.2±503.7	1,123.3±589.6	0.156
Events			
Hypertension	12 (50)	21 (63.6)	0.303
Hypotension	13 (54.2)	17 (51.5)	0.843
Arrthymia	4 (16.7)	4 (12.1)	0.626
Vasopressor intermittent bolus	16 (66.7)	19 (57.6)	0.486
Vasopressor infusion	4 (16.7)	2 (6.1)	0.198
Emergence			
Extubation after operation (n=24)	8 (33.3)	16 (48.5)	0.253
Post-operative			
Extubation at ICU (n=33)	16 (66.7)	17 (51.5)	0.253
Time to extubation at ICU (min)	872.4±593.3	601.6±473.9	0.156

The data are presented as mean±standard deviation or n (%), P value<0.05 indicates statistical significance.

The spectral edge frequency 95% (SEF95%) of both the BS and non-BS groups tended to decrease after carotid clamping, as indicated in Table 3. However, the two groups had no significant difference regarding MED or SEF95% before and after the clamping. Similarly, neither group significantly differed between the left and right MED or SEF95%. After carotid clamping, the BS group had an average BSR of 36.0±20.4 (right) and 36.3±20.6 (left), but this difference was not statistically significant. In contrast, the non-BS group did not exhibit any burst suppression pattern.

**Table 3.** The EEG data before and the average of EEG data during the carotid clamp time.

Variables		Group BS (n=24)			Group Non-BS (n=33)		
Phase of carotid clamp		Before	During	p-value	Before	During	p-value
MED (Hz.)	Right side	1.8±0.5	1.8±0.6	0.342	1.8±0.8	1.9±0.7	0.290
	Left side	1.8±0.6	1.8±0.5	0.859	1.9±0.5	1.8±0.7	0.284
SEF95% (Hz.)	Right side	9.2±2.9	8.8±2.0	0.311	9.5±4.2	9.3±2.8	0.585
	Left side	9.2±2.7	8.9±1.9	0.541	10.2±4.3	9.3±2.8	0.207
BS (%)	Right side	0	36.0±20.4	-	0	0	-
	Left side	0	36.3±20.6	-	0	0	-

The data are presented as mean±standard deviation or n (%), P value<0.05 indicates statistical significance.

The incidence of hypertension, hypotension, and arrhythmias did not show a statistically significant difference between the two groups. Additionally, there was no notable difference in the incidence of vasopressor intermittent boluses or vasopressor infusions. Following the operation, eight patients (33.3%) in the BS group and sixteen (48.5%) in the non-BS group were awake and extubated. Most patients in both groups were intubated and transferred to the neurosurgical intensive care unit. There was no significant difference in extubation time for patients who were initially unable to extubate between the two groups (BS group 872.4±593.3 min. vs. non-BS group 601.6±473.9 min). One patient experienced a mild neurological deficit. No deaths were reported one month after the operation ( Table 4).

**Table 4.** Clinical outcomes.

Variables	Group BS (n=24)	Group Non-BS (n=33)	p-value
Postoperative outcomes			
TIA	1 (4.2)	3 (9.1)	0.472
RIND	1 (4.2)	1 (3)	0.818
Mild neurological deficit	0 (0)	1 (3)	0.39
Length of ICU stay (day)	1.7±1.1	1.4±1.1	0.418
Length of hospital stay (day)	12.5±13.1	14.5±11.0	0.54
GOS at discharge=5	22 (91.7)	29 (87.9)	0.645
GOS at 1 month=5	23 (95.8)	28 (84.8)	0.182

The data are presented as mean±standard deviation or n (%), P value<0.05 indicates statistical significance. TIA=transient ischemic attack; RIND=reversible ischemic neurological disability; GOS=Glasgow Outcome Scale.

## Discussion

This study aimed to determine the amount of thiopental required to induce burst suppression patterns on intraoperative EEG monitoring in patients undergoing carotid endarterectomy without a shunt. The main findings indicated that not all patients achieved burst suppression despite the intention to maximize cerebral protection through a continuous thiopental infusion and titrated intravenous administration. Patients who received significantly higher doses of thiopental (26.3±10.1 mg/kg/hr) were likelier to achieve burst suppression on EEG. However, no significant difference was observed in postoperative outcomes between the burst suppression (BS) and non-burst suppression (non-BS) groups. Currently, limited data is available on the efficacy and optimal dosage of thiopental for inducing pharmacological burst suppression to prevent perioperative stroke during selective shunting in CEA.

Barbiturates, such as thiopental, are commonly used to prevent cerebral ischemia during cerebrovascular surgery. Thiopental is a fast-acting, short-duration barbiturate anesthetic that may exert its neuroprotective effects through various mechanisms, including antioxidant activity, GABA-ergic activity, stimulation of protein synthesis, removal of free radicals, and modulation of excitatory synaptic neurotransmission via adenosine. ^19^ Animal studies have shown that barbiturates can decrease brain oxygen demand and the size of cerebral infarction. ^20^^–^^22^ In cerebrovascular procedures that require temporary clips, such as extracranial-intracranial bypasses, carotid endarterectomies, and aneurysm clipping, barbiturates have been demonstrated to reduce the cerebral metabolic rate of oxygen and increase blood flow to ischemic regions. ^23^^–^^25^

The thiopental dose required for EEG burst suppression patterns during cerebrovascular surgery can vary depending on several factors, including variability in monitoring and assessing burst suppression levels among healthcare providers. Sreedhar and Gadhinglajkar ^26^ have reviewed several dosing regimens of thiopental for cerebrovascular surgery, including a bolus dose (4 mg/kg), a low dose followed by IV infusion (1 to 3 mg/kg IV followed by 0.06 to 0.2 mg/kg/min), and a high dose followed by infusion (loading 25 to 50 mg/kg followed by 2 to 10 mg/kg/hr).

The initial bolus doses of thiopental used in our study did not result in EEG burst suppression for most patients, which differs from the findings of Ramesh VJ. ^27^ According to Ramesh VJ, almost all patients who received a bolus dose of 3 to 5 mg/kg experienced EEG burst suppression with a BSR greater than 25%. The initial bolus doses of thiopental only resulted in temporary suppression durations, consistent with previous studies by Moffat et al. ^28^ and Gelb et al., ^29^ which provided limited cerebral protection during the intraoperative period.

Our study used a continuous thiopental infusion to maintain EEG burst suppression during the carotid cross-clamp procedure. We administered a high dose of thiopental, similar to previous studies by McConkey PP et al. ^30^ and Frawley JE et al. ^31^ However, not all patients in our study achieved EEG burst suppression, unlike the abovementioned studies where incremental bolus doses of thiopental were titrated. Nonetheless, none of our patients experienced a significant period of ischemia, as defined by a decrease in SEF95% to 50% of the baseline. ^32^

EEG-confirmed burst suppression is widely recognized as the preferred indicator of cerebral protection during cerebrovascular surgery with barbiturate therapy.

Our institute protocol primarily relied on electroencephalography (EEG) to assess burst suppression. While intraoperative monitoring of burst suppression is commonly performed using EEG or Bispectral Index (BIS), ^33^^,^^34^ it has been observed that BIS-derived Burst Suppression Ratio (BSR) may underestimate the duration of EEG suppression, thereby reducing sensitivity in detecting burst suppression. ^35^ We directly visualized the EEG trace to ensure a more accurate and real-time evaluation of raw EEG changes and BSR values across both brain hemispheres. This approach enabled us to detect potential cerebral ischemia and determine cerebral protection levels.

However, we recognize the significance of monitoring cerebral hemodynamics and function, ^36^ including sensory-evoked potential (SEP), motor-evoked potential (MEP), and near-infrared spectroscopy (NIRS). ^37^ In 2014, the institute began implementing sensory-evoked potential monitoring (SEP) and motor-evoked potential (MEP) for spinal surgery. However, these methods are not routinely employed for carotid endarterectomy, partly due to the impact of burst suppression on EEG functional assessment. ^38^^,^^39^ Additionally, cerebral oximetry was first introduced in 2022.

The study found that older patients were more likely to achieve burst suppression on EEG with thiopental therapy, potentially due to age-related factors. ^40^ Although not all patients achieved burst suppression, the BS and non-BS groups had favorable clinical outcomes without significant perioperative complications. The BS group did have a more extended postoperative intubation period. However, their Glasgow Outcome Scale was not significantly different from the non-BS group at discharge or one month after surgery. These outcomes could be attributed to barbiturate therapy, as research on rats suggests that EEG burst suppression may not be necessary for maximum cerebral protection.

The dosage of thiopental that does not lead to EEG burst suppression may impact cerebral protection during carotid endarterectomy. However, thiopental-induced cerebral protection is only one of several strategies for cerebral protection. Other factors, such as the degree of stenosis, pre-existing medical conditions, and perioperative risks associated with carotid endarterectomy, influence patient outcomes. ^41^

Limitations of this study include the fact that it exclusively focused on the dosage of thiopental and its effects on intraoperative EEG monitoring as a direct tool for evaluating brain electrical activity during carotid endarterectomy. In the non-BS group, our investigation revealed that both anesthesiologists and nurse anesthetists expressed concern about hypotension and increased thiopental dose, which may lead to delayed extubation in the postoperative period. Consequently, a decision may have been made to avoid increasing the dosage and frequency of thiopental enough to induce EEG burst suppression. As a result, the insufficient thiopental dosage hinders the achievement of EEG suppression, and leaving the exact dosage needed for EEG burst suppression in this patient group undetermined. It is essential to acknowledge that EEG only provides information on brain electrical activity and does not directly measure cerebral blood flow or cerebral oxygen saturation (SjVO _2_). Therefore, relying solely on EEG may not accurately reflect the actual level of cerebral protection. Multimodal intraoperative monitoring techniques, such as transcranial Doppler (TCD) ultrasound, somatosensory evoked potentials, and cerebral oxygen saturation monitoring, are recommended for a more comprehensive patient status assessment. Additionally, this study did not assess the thiopental serum levels for barbiturate coma treatment. The retrospective cohort design also limited the quality of data collection. The limited number of patients has additional challenges in analyzing patient outcomes. Therefore, a prospective randomized study is recommended to investigate further the role of intraoperative monitoring and barbiturate therapy in optimizing cerebral protection during carotid endarterectomy.

In conclusion, thiopental at a dosage of 26.3±10.1 mg/kg/hr can induce burst suppression on intraoperative EEG during carotid endarterectomy, potentially providing cerebral protection. However, a cautious approach is recommended when using thiopental to induce EEG burst suppression during carotid clamping. While barbiturate-induced cerebral protection is still considered to have a role, the necessity of EEG burst suppression for cerebral protection requires further investigation. Considering the limitations of our retrospective study, the recommended strategy should be individualized for each patient, considering available resources based on institutional protocols. Further evidence from medical studies, neurophysiology studies, and mathematical modeling is needed to gain a comprehensive understanding of burst suppression and its potential therapeutic applications. Therefore, it is crucial to carefully evaluate the potential benefits and risks of using burst suppression in clinical practice and identify the optimal perioperative settings where it may be beneficial.

## Data Availability

Figshare: DATA of ThioBS_CEA, https://doi.org/10.6084/m9.figshare.22132898.v1. ^42^ This project contains the following underlying data:
- f1000-ThioBSR_2_21_23.xlsx Data are available under the terms of the Creative Commons Attribution 4.0 International license (CC-BY 4.0).
