# Supplementary material for: The dosage of thiopental as pharmacological cerebral protection during non-shunt carotid endarterectomy: A retrospective study
Source: F1000Res. 2023 Dec 11;12:381. Originally published 2023 Apr 11. [Version 3] doi: 10.12688/f1000research.131838.3 (PMC10748806; doi:10.12688/f1000research.131838.3)
Supplement: Peer review history

# Reviewer report: Reviewer response for version 3

**DOI:** 10.5256/f1000research.159803.r229098

**Published:** 2023-12-23

**Authors:** Antonello Penna

**Affiliations:** 1 Department of Anesthesia, Clinical Hospital & Center for Advanced Clinical Research (CICA), Faculty of Medicine, University of Chile, Santiago, Chile

Dear authors,

 The authors seem to respond all comments raised by the reviewer. The contents of this manuscript have become much clearer. The data obtained in this study would be of clinical value. Thank you very much.

Is the work clearly and accurately presented and does it cite the current literature?

Partly

If applicable, is the statistical analysis and its interpretation appropriate?

Yes

Are all the source data underlying the results available to ensure full reproducibility?

Yes

Is the study design appropriate and is the work technically sound?

Partly

Are the conclusions drawn adequately supported by the results?

Partly

Are sufficient details of methods and analysis provided to allow replication by others?

Partly

Reviewer Expertise:

Anesthesiology and EEG intraopertive monitoring

I confirm that I have read this submission and believe that I have an appropriate level of expertise to confirm that it is of an acceptable scientific standard.

# Reviewer report: Reviewer response for version 2

**DOI:** 10.5256/f1000research.152260.r205938

**Published:** 2023-10-02

**Authors:** Antonello Penna

**Affiliations:** 1 Department of Anesthesia, Clinical Hospital & Center for Advanced Clinical Research (CICA), Faculty of Medicine, University of Chile, Santiago, Chile

Dear authors, I have carefully read your manuscript in which the aim was to study the optimal dose of barbiturates, such as thiopental, to induce patterns in the EEG of burst suppression in patients undergoing carotid endarterectomy with a non-shunt technique. The article is interesting and possibly useful for the clinical practice of many centers. I understand that currently, for example, in our center, it is preferred to monitor with sensory or motor potentials in vascular neurosurgeries and in order not to affect the monitoring, we do not induce suppression and, finally, perform an early extubation after the cessation of propofol perfusion. However, knowing the dose needed to reach suppression is a necessary and interesting fact, but I suggest making the following modifications to the manuscript:

 Feedback:

1. A limitation of the study is its retrospective design. It is not clear why some patients reached burst suppression while in others it was not reached. This fact could affect the recommended dose of thiopental to achieve burst suppression. In the group of patients in which burst suppression was not generated, it is unknown what dose is necessary to achieve suppression. That should be discussed in the limitations.
2. The factors that could explain why in one case burst suppression was achieved and in others no, it was not explored. At first, one possible explanation was that some clinicians decided to increase the dose of thiopental until they reached suppression while others did not. Therefore, it would be interesting to analyze whether the operators who achieved suppression were the same and those who were others. Another option may have been difficulty maintaining blood pressure above 10% during clamp. Perhaps patients who achieved burst suppression were more likely to reach that goal with fewer doses of vasopressor, while in those who did not reach it was more difficult to reach that goal with higher doses of vasopressors. I suggest incorporating both analyses into the results section and then discussing those analyses.
3. In the first sentence of the discussion, it is said that one aim was to evaluate the outcomes of patients, but that aim was not the primary one and was not mentioned at the end of the introduction. I suggest removing it in the discussion sentence. Just mention it as a secondary outcome and emphasize that with the number of patients it is difficult to reach a conclusion.
4. Page 4: this sentence is not well understood: "thiopental (5 mg/kg) was given intravenously (IV) followed by continuous infusion of 10 mg/kg/hr with an additional 50 mg titrated intravenously". The additional 50 mg was always given, at what time?
5. This sentence is confusing, please review: "glycopyrrolate 0.2 mg for each 1.0 mg of neostigmine or atropine 0.02 mg/kg and neostigmine 0.02 to 0.07 mg/kg".
6. In Table 2, the values of Sevoflurane/Desflurane (% end tidal), were during the clamp? Or at the end? Please specify.
7. Please specify the unit when talking about burst suppression or MED. For example, in the sentence "BSR of 36.020.4 (right) and 36.320.6 (left)" and in Table 3, is % or min?

Is the work clearly and accurately presented and does it cite the current literature?

Partly

If applicable, is the statistical analysis and its interpretation appropriate?

Yes

Are all the source data underlying the results available to ensure full reproducibility?

Yes

Is the study design appropriate and is the work technically sound?

Partly

Are the conclusions drawn adequately supported by the results?

Partly

Are sufficient details of methods and analysis provided to allow replication by others?

Partly

Reviewer Expertise:

Anesthesiology and EEG intraopertive monitoring

I confirm that I have read this submission and believe that I have an appropriate level of expertise to confirm that it is of an acceptable scientific standard, however I have significant reservations, as outlined above.

# Reviewer report: Reviewer response for version 2

**DOI:** 10.5256/f1000research.152260.r183586

**Published:** 2023-07-19

**Authors:** Masahiko Kawaguchi

**Affiliations:** 1 Department of Anesthesiology and Division of Intensive Care, Nara Medical University, Nara, Japan

Thank you very much for revising the manuscript, which was originally submitted. The authors seem to respond all comments raised by the reviewer. The contents of this manuscript have become much clearer. Although there are several limitations of this study, the data obtained in this study would be of clinical value. Thank you very much.

Is the work clearly and accurately presented and does it cite the current literature?

No

If applicable, is the statistical analysis and its interpretation appropriate?

Yes

Are all the source data underlying the results available to ensure full reproducibility?

Yes

Is the study design appropriate and is the work technically sound?

Partly

Are the conclusions drawn adequately supported by the results?

Partly

Are sufficient details of methods and analysis provided to allow replication by others?

Yes

Reviewer Expertise:

Neuroanaesthesia, neuromonitoring

I confirm that I have read this submission and believe that I have an appropriate level of expertise to confirm that it is of an acceptable scientific standard.

# Reviewer report: Reviewer response for version 1

**DOI:** 10.5256/f1000research.144721.r169287

**Published:** 2023-05-19

**Authors:** Masahiko Kawaguchi

**Affiliations:** 1 Department of Anesthesiology and Division of Intensive Care, Nara Medical University, Nara, Japan

The authors retrospectively evaluated the optimal dosage of thiopental required to induce burst suppression during non-shunt carotid endarterectomy. As an analysis of 57 patients, the authors concluded the optimal dosage of thiopental required to achieve burst suppression on intraoperative EEG was 26.3+10.1 mg/kg/hr. This is interesting clinical data. However, there are several concerns which should be clarified.

 In all patients, non-shunt technique was used. How did the authors evaluate that these patients would not require shunt during carotid clamping? Were there patients in which poor collateral flow was expected during the carotid clamping? Please mention the method of preoperative assessment.

 Based on the results of this study, the outcome of the patients was similar. In addition, previous animal study indicated that burst suppression is not necessary to achieve neuroprotective effect of thiopental (Schmid-Elsaesser *et al. *(1999) ^1^). So, this reviewer doubts that burst suppression is really required during the clamping of carotid artery. The authors should mention the rationale to get burst suppression on EEG. Please add the data that burst suppression would be required to have neuroprotective effects. This can be a limitation of this study. 

 Was any monitoring of cerebral hemodynamics and function performed, including near-infrared spectroscopy (NIRS), motor evoked potential or sensory evoked potential? Strategy to identify the function during carotid clamp is also important to prevent postoperative neurological dysfunction. Under the burst suppression on EEG, assessment of function can be disturbed.  

 Finally, based on the data of this study, what did the authors recommend as a strategy to use thiopental during carotid clamping? ****Considering the necessity of burst suppression for neuroprotection, the conclusion should be carefully drawn.

Is the work clearly and accurately presented and does it cite the current literature?

No

If applicable, is the statistical analysis and its interpretation appropriate?

Yes

Are all the source data underlying the results available to ensure full reproducibility?

Yes

Is the study design appropriate and is the work technically sound?

Partly

Are the conclusions drawn adequately supported by the results?

Partly

Are sufficient details of methods and analysis provided to allow replication by others?

Yes

Reviewer Expertise:

Neuroanaesthesia, neuromonitoring

I confirm that I have read this submission and believe that I have an appropriate level of expertise to confirm that it is of an acceptable scientific standard, however I have significant reservations, as outlined above.

## References

[1] : EEG burst suppression is not necessary for maximum barbiturate protection in transient focal cerebral ischemia in the rat. *Journal of the Neurological Sciences* .1999;162(1) : 10.1016/S0022-510X(98)00300-1 14-19 10.1016/S0022-510X(98)00300-1 10064163

# Response

**Published:** 2023-06-27

**Authors:** pimwan sookplung

Dear Dr. Masahiko Kawaguchi.

 Thank you very much for taking the time to contribute to the opportunity to improve the research article. I hope our revised paper will be appreciated by you.

 Regards,

 Pimwan Sookplung, MD.

 Department of Anesthesiology, Neurological Institute of Thailand, Bangkok, Thailand

**Comments:**

**1.In all patients, non-shunt technique was used. How did the authors evaluate that these patients would not require shunt during carotid clamping? Were there patients in which poor collateral flow was expected during the carotid clamping? Please mention the method of preoperative assessment.**

**Response:** To determine whether patients would require a shunt during carotid clamping, the neurosurgeon likely employed preoperative investigation by Carotid Duplex Ultrasound and Magnetic resonance angiography (MRA) to assess the carotid bifurcation disease, the degree of stenosis, and evaluate the adequacy of collateral circulation. Conventional angiography was performed only in some cases to delineate the actual stenosis segment or to evaluate the carotid disease and status of the collateral circulation. Patients with poor collateral flow will undergo a specific intraoperative assessment using the "Backflow technique." If the assessment reveals insufficient blood flow, an intraluminal shunt will ensure adequate blood supply during carotid clamping.

**2. Based on the results of this study, the outcome of the patients was similar. In addition, previous animal study indicated that burst suppression is not necessary to achieve neuroprotective effect of thiopental (Schmid-Elsaesser et al. (1999)1). So, this reviewer doubts that burst suppression is really required during the clamping of carotid artery. The authors should mention the rationale to get burst suppression on EEG. Please add the data that burst suppression would be required to have neuroprotective effects. This can be a limitation of this study.**

**Response:** The rationale for inducing burst suppression is based on its theorized potential neuroprotective effects. Burst suppression reduces metabolic demand by decreasing neuronal activity and electrical signaling, reduction in cerebral blood flow (CBF), and preserving limited energy resources during critical situations. Reducing metabolic requirements helps conserve oxygen supply, mitigating the risk of oxygen deprivation and preventing additional brain damage.

 Although Robert Schmid-Elsaesser (1999) indicates that EEG burst suppression is not necessary for maximum cerebral protection. However, animal studies may not be conclusive in humans due to inter-species differences in burst suppression effects and differences in physiology and the human clinical context. Doyle PW ^^(1999) suggests that if the flow-metabolism coupling is intact, complete EEG burst suppression (100% burst suppression) may provide more cerebral protection than 50% burst suppression. However, the study did not evaluate the cerebral protection effects. Thus, human studies regarding burst suppression and cerebral protection effect are still needed.

**3. Was any monitoring of cerebral hemodynamics and function performed, including near-infrared spectroscopy (NIRS), motor evoked potential or sensory evoked potential? Strategy to identify the function during carotid clamp is also important to prevent postoperative neurological dysfunction. Under the burst suppression on EEG, assessment of function can be disturbed.**

**Response: **During the study period from January 2009 to December 2019, our institute primarily relied on electroencephalography (EEG) for assessing burst suppression. However, we recognize the significance of monitoring cerebral hemodynamics and function, including sensory-evoked potential (SEP), motor-evoked potential (MEP), and near-infrared spectroscopy (NIRS).

 In 2014, the institute began implementing sensory-evoked potential monitoring (SEP) and motor-evoked potential (MEP) for spinal surgery. However, these methods are not routinely employed for carotid endarterectomy, partly due to the impact of burst suppression on EEG functional assessment. Additionally, cerebral oximetry was first introduced in 2022.

**4. Finally, based on the data of this study, what did the authors recommend as a strategy to use thiopental during carotid clamping? Considering the necessity of burst suppression for neuroprotection, the conclusion should be carefully drawn.**

**Response:** Based on our study's data, a cautious approach is recommended when using thiopental to induce EEG burst suppression during carotid clamping. While barbiturate-induced cerebral protection is still considered to have a role, the necessity of EEG burst suppression for cerebral protection requires further investigation. Considering the limitations of our retrospective study, the recommended strategy should be individualized for each patient, considering available resources based on institutional protocols. Further evidence from medical studies, neurophysiology studies, and mathematical modeling is needed to gain a comprehensive understanding of burst suppression and its potential therapeutic applications. Therefore, it is crucial to carefully evaluate the potential benefits and risks of using burst suppression in clinical practice and identify the optimal perioperative settings where it may be beneficial.
